# Outcomes of Gastrointestinal Polyps Resected Using Underwater Endoscopic Mucosal Resection (UEMR) Compared to Conventional Endoscopic Mucosal Resection (CEMR)

**DOI:** 10.7759/cureus.11485

**Published:** 2020-11-14

**Authors:** Mohamad Awf Mouchli, Shravani Reddy, Chirstopher Walsh, Adil Mir, Lindsey Bierle, Vikas Chitnavis, Paul Yeaton, Mohammad Shakhatreh

**Affiliations:** 1 Gastroenterology, Cleveland Clinic, Cleveland, USA; 2 Internal Medicine, Carilion Clinic, Virginia Tech Carilion School of Medicine, Roanoke, USA; 3 Internal Medicine, Carilion Clinic, Virginia Tech Carilion School of Medicine, Ronaoke, USA; 4 Gastroenterology and Hepatology, Carilion Clinic, Virginia Tech Carilion School of Medicine, Roanoke, USA

**Keywords:** polypectomy, piecemeal, recurrence, underwater endoscopic mucosal resection (uemr), conventional endoscopic mucosal resection (cemr)

## Abstract

Objective

Underwater endoscopic mucosal resection (UEMR) is reported to be superior to conventional endoscopic mucosal resection (CMER) for the complete resection of large polyps and may offer increased procedural efficiency.

Aims

To compare recurrence rates and adverse events between UEMR and CEMR and define risk factors related to recurrence. Also, to assess recurrence rates in piecemeal endoscopic mucosal resection (EMR) based on the number of pieces resected.

Methods

We identified all patients with large polyps treated using the UEMR technique at Carilion Clinic, Roanoke, VA, USA between January 1, 2014 and December 31, 2017 with follow-up through October of 2018. We matched the UEMR patients with patients treated using the CEMR technique (1:2 matching, respectively). The Kaplan-Meier curve was used to estimate the cumulative risks of polyp recurrence. The Cox proportional hazard analysis was used to assess risk factors for developing polyp recurrence.

Results

Sixty-eight patients (mean age: 63.4 ± 12.5 years; 52.9% males) with polyps removed using the UEMR technique (Group 1) were matched with 122 patients (mean age: 64.4 ± 10.0 years; 51.6% males) who had polyps removed using CEMR (Group 2). Polyps resected in fewer pieces (≤ 3) had lower recurrence rates compared to the ones resected in >3 pieces. Right colon polyps removed using UEMR had a lower recurrence rate compared to right colon polyps resected using CEMR. Polyp size and a high degree of dysplasia were associated with a high risk of polyp recurrence after resection. Completing advanced endoscopy training was also associated with a lower risk of recurrence.

Conclusion

UEMR had a lower recurrence rate compared with CEMR for right colon polyps. Factors associated with recurrence included the degree of training, high-grade dysplasia, and polyp size.

## Introduction

Colorectal cancer was reported by the American Cancer Society to be the third most commonly diagnosed cancer among males and females in 2017 with steadily decreasing mortality [1]. It usually begins as a polyp, and Vogelstein et al. first described the progression of colorectal cancer from the growth of pre-malignant polyps via a stepwise accumulation of mutations in tumor suppressor genes and oncogenes [2]. Therapeutic colonoscopy to remove polyps is reported to be associated with a reduction in colorectal cancer-related mortality [3-4].

Conventional endoscopic mucosal resection (CEMR) is the traditional method to remove large polyps which typically utilizes submucosal injection (the “inject-and-cut” technique) to lift the polyp to ensure complete resection. This technique was first described in humans in 1994 by Yokota et al. [5]. It is associated with a 12.2% to 55% recurrence rate at the polypectomy site if resected in a piecemeal fashion [6] and with a 2% to 24% risk of intraprocedural bleeding [7]. Adenoma characteristics (i.e., histology, size, multiplicity, and location) and incomplete resection are important indicators of recurrence after polypectomy [8].

In recent years, underwater endoscopic mucosal resection (UEMR) was developed as an alternative technique for the removal of large colorectal polyps. This technique was first described by Binmoeller et al. as a technique that utilizes water immersion to allow for better visualization of a target lesion and “float” the mucosa/submucosa away from the deeper muscularis propria in order to perform endoscopic mucosal resection (EMR) without submucosal injection [9]. A few studies have reported this technique to be superior to CEMR for complete resection of large colorectal polyps with fewer adverse events and residual polyp rate ranging between 2.0% to 8.8% [9-11]. UEMR is reported to be safe, except for two reported cases of perforation, one of which occurred in the retroflex position [12-13]. Siau et al. reported that piecemeal resection, recurrent polyp, female gender, and difficult access are predictors of post-UEMR polyp recurrence [14].

Though several studies have illustrated the safety and superiority of underwater polypectomy in resecting colon polyps, we aimed to evaluate our local UEMR experience in removing upper gastrointestinal tract and colon polyps and compare recurrence rates and adverse events to CEMR. Our secondary goals were to define risk factors related to recurrence and to compare recurrence rates in patients undergoing piecemeal resection based on the number of pieces resected.

## Materials and methods

Study population

In this retrospective study approved by the Carilion Clinic Institutional Review Board (IRB) (approval #2496), we reviewed the esophagogastroduodenoscopy (EGD) and colonoscopy data, as well as the pathology reports for patients who were seen at Carilion Clinic for EGD or colonoscopy with submucosal injection using CPT codes 43211 and 45381. We also reviewed some endoscopy reports manually to identify the rest of the UEMR cases done before using CPT codes 43211 and 45381 between January 1, 2014 through December 31, 2017, with follow-up through October of 2018.

In our practice, three providers use UEMR and six providers use CEMR to resect large polyps. We identified all patients who underwent UEMR and met the inclusion criteria and compared them to randomly selected controls who underwent conventional EMR at a 1:2 ratio, respectively, over the same time frame in order to compare the rates of complete resection and evaluate risk factors for incomplete resection. All patients had at least one surveillance examination following the index polypectomy. Surveillance examinations were performed only for follow-up and were not done in response to clinical symptoms.

We included all patients ≥ 18 years of age diagnosed with large (≥ 10 millimeters (mm)) polyps (large upper hyperplastic polyp, tubular adenomas, villous adenomas, sessile serrated adenomas, traditional serrated adenomas, and cancerous polyps) requiring EMR between January 1, 2014 and December 31, 2017. Patients were followed through October 2018. Patients with polyps requiring endoscopic submucosal dissection (ESD) or full-thickness resection (FTR) were excluded from the study. We also excluded patients with Crohn’s disease, nodular Barrett’s esophagus, patients with other polypoid lesions requiring EMR (carcinoid tumors, leiomyomas, benign tissues, inflammatory polyps, etc.), and patients who did not have surveillance endoscopy following the index polypectomy. We identified all patients from this cohort who had polyps (one polyp per patient) resected using UEMR (n = 89) and matched them to 177 patients who had polyps removed using CEMR. Clinical and pathological features of high-risk polyps (i.e., size, histology, site, and degree of dysplasia), number and timing of surveillance endoscopies, and recurrent polyp clinical and pathological features (i.e., size, histology, and degree of dysplasia) were collected via a one-time data extraction from the electronic medical record. Data were abstracted into REDCap ® (Research Electronic Data Capture) software (Vanderbilt University, Nashville, TN). Subjects who underwent CEMR were randomly selected from a pool of patients matched to the UEMR group based on age, gender, and year that the index polyp was removed.

Saline and Eleview® submucosal injectable composition were used in all CMER cases. It was also used in a few UEMR cases after water immersion if the polyp was not completely engaged.

A recurrent polyp was classified as a same site histological recurrence if it arose in the region in which the polyp had been removed after any subsequent endoscopy. The surveillance endoscopy protocol after the removal of large lesions was based on the individual endoscopist’s practice and preference. For the most part, patients with polyps with high-grade dysplasia removed in piecemeal underwent repeat endoscopic examinations in three to six months. Endoscopic evaluation was done after six to 12 months for polyps with low-grade to no dysplasia removed in a piecemeal fashion. Polyps removed en bloc were assessed after three years for recurrence or sooner based on the individual endoscopist’s evaluation during the index procedure.

Statistical analysis

We reported the data as mean (± standard deviation (SD)), median (interquartile range (IQR)), ranges, and categorical variables. We used Chi-square/Fisher's exact test for categorical and the t-test or Wilcoxon signed-rank test for continuous variables. Estimates of the rate of polyp recurrence and cumulative incidence of polyp recurrence were estimated by using the Kaplan-Meier survival curve with a log-rank test. To identify risk factors associated with polyp recurrence, we performed the univariate time-to-event analysis with Cox proportional regression models and robust estimates of variance. We included variables with p < 0.05 on univariate analysis in a multivariate Cox proportional hazard analysis to identify independent risk factors associated with polyp recurrence. We used JMP®, version 10 for Windows (SAS Institute Inc., Cary, NC, USA) to conduct all statistical analyses. 

## Results

Patient eligibility

**Figure 1:**
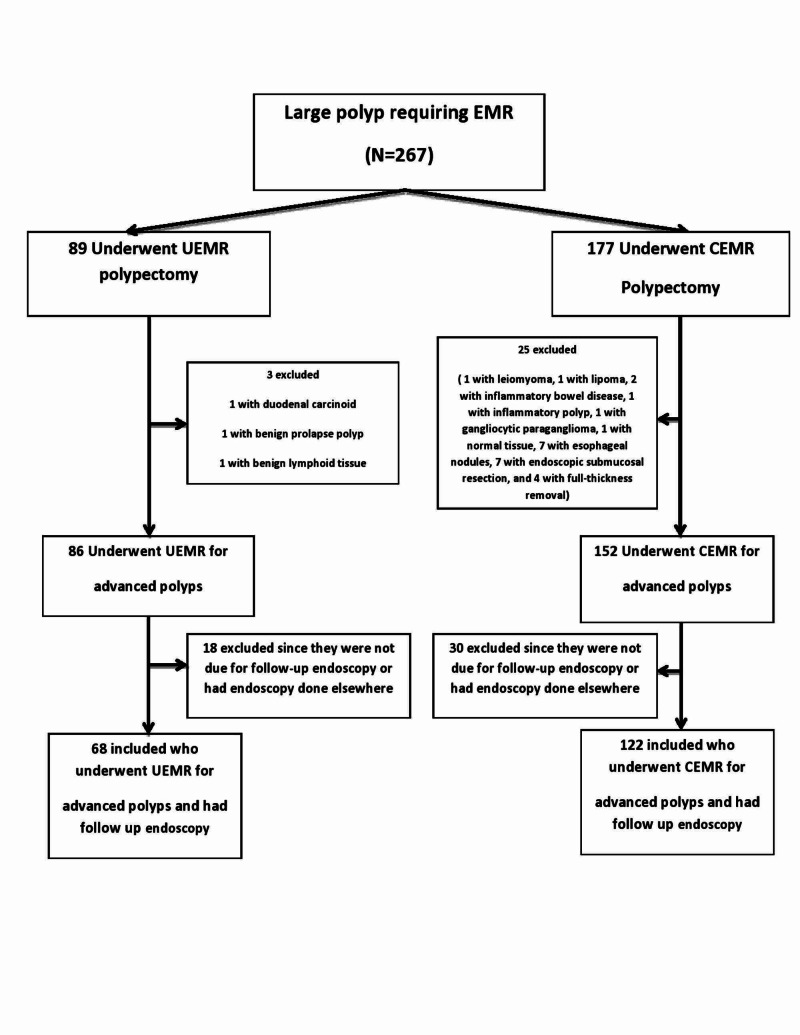
Flowchart of the study CEMR: conventional endoscopic mucosal resection; EMR: endoscopic mucosal resection; UEMR: underwater endoscopic mucosal resection

A total of 276 patients with 276 large polyps requiring UEMR or CEMR were studied. Eight-nine underwent UEMR and 177 underwent CEMR. After excluding 22 patients in the UEMR group and 55 patients in the CEMR group since they did not meet inclusion criteria and were not due for a surveillance endoscopy after this index polypectomy, 190 patients were evaluated (Figure 1). 

Results

Sixty-eight patients who underwent UEMR were identified. These 68 patients were compared to a randomly selected cohort of 122 of the CEMR patients. UEMR cases were mainly done by faculty who completed advanced training (94.1% vs 43.4%, p < 0.01), had more polyps removed in piecemeal fashion (64.2% vs 38.0%, p < 0.01), and had less number of pieces when removed in piecemeal fashion (47.0% vs 78.8%; p < 0.01). Early bleeding (within 48 hours of polypectomy not including intraprocedural bleeding) occurred in two (2.94%) patients of the UEMR group compared to 0 (0%) in the CEMR group. Delayed bleeding from the polypectomy site (up to 30 days) was not significantly different between the two groups (4.4% in the UEMR group and 4.9% in the CEMR group, p = 0.054). Other findings are shown in Table 1. 

**Table 1:** Clinical and Demographic Characteristics of Patients with Large Polyps Removed Using UEMR vs. CEMR Techniques CEMR: conventional endoscopic mucosal resection; EMR: endoscopic mucosal resection; IQR: interquartile range; SD: standard deviation; UEMR: underwater endoscopic mucosal resection

	UEMR (N=68)	CEMR (N=122)	p-value
Demographics			
Age at resection (mean ± SD)	63.37 ± 12.46	64.43 ± 10.00	0.985
Male gender, n (%)	36 (52.94%)	63 (51.64%)	0.897
Advanced training, n (%)	64 (94.12%)	53 (43.44%)	< 0.001
Features of the resected polyps for patients with follow-up			
Polyp size (< 2 cm), n (%)	32 (47.06%)	60 (49.18%)	0.203
Polyp size (2 - 4 cm), n (%)	32 (47.06%)	49 (40.16%)	
Polyp size (> 4 cm), n (%)	4 (5.88%)	13 (10.66%)	
Number of polyps resected at index endoscopy (median, IQR)	2.0, 1 - 4	2.0, 1 - 3	0.004
Number of endoscopies required to remove the polyp (median, IQR)	2.0, 1 - 4.25	2.0, 1 - 3	0.037
Polyp location			
Gastric polyps, n (%)	3 (4.41%)	13 (10.66%)	0.061
Duodenal polyps, n (%)	4 (5.88%)	16 (13.11%)	
Colon polyps, n (%)	61 (89.71%)	93 (76.23%)	
Right colon, n (%)	48 (78.69%)	55 (59.14%)	0.049
Additional treatments			
Piecemeal removal, n (%)	43 (64.18%)	46 (38.02%)	< 0.001
Injection-assisted EMR, n (%)	10 (14.93%)	122 (100.0%)	< 0.001
Flat/sessile, n (%)	65 (95.59%)	109 (90.08%)	0.161
Polyp pathology			
Tubular adenoma, n (%)	39 (59.09%)	57 (48.72%)	0.137
Villous adenoma, n (%)	12 (18.18%)	20 (17.09%)	
Hyperplastic polyp, n (%)	2 (3.03%)	15 (12.82%)	
Sessile serrated adenoma, n (%)	13 (19.70%)	24 (20.51%)	
Traditional serrated adenoma, n (%)	0 (0.0%)	1 (0.85%)	
Superficial adenocarcinoma, n (%)	2 (2.94%)	3 (2.50%)	0.656
High-grade dysplasia, n (%)	6 (8.82%)	11 (11.67%)	
Low-grade dysplasia, n (%)	25 (36.76%)	34 (28.33%)	
No/unknown dysplasia, n (%)	35 (51.47%)	69 (57.50%)	
Device used			
Hot snare, n (%)	46 (67.65%)	46 (37.70%)	< 0.001
Cold snare, n (%)	1 (1.47%)	12 (9.84%)	
Non-specified snare, n (%)	21 (30.88%)	64 (52.64%)	
Polyp recurrence			
Polyp recurrence at the polypectomy site, n (%)	13 (19.12%)	33 (27.05%)	0.215
Complications			
Early bleeding from polypectomy site, n (%)	2 (2.94%)	0 (0.00%)	0.054
Delayed bleeding from polypectomy site, n (%)	3 (4.41%)	6 (4.92%)	

Polyp recurrence after UEMR and CEMR

**Figure 2:**
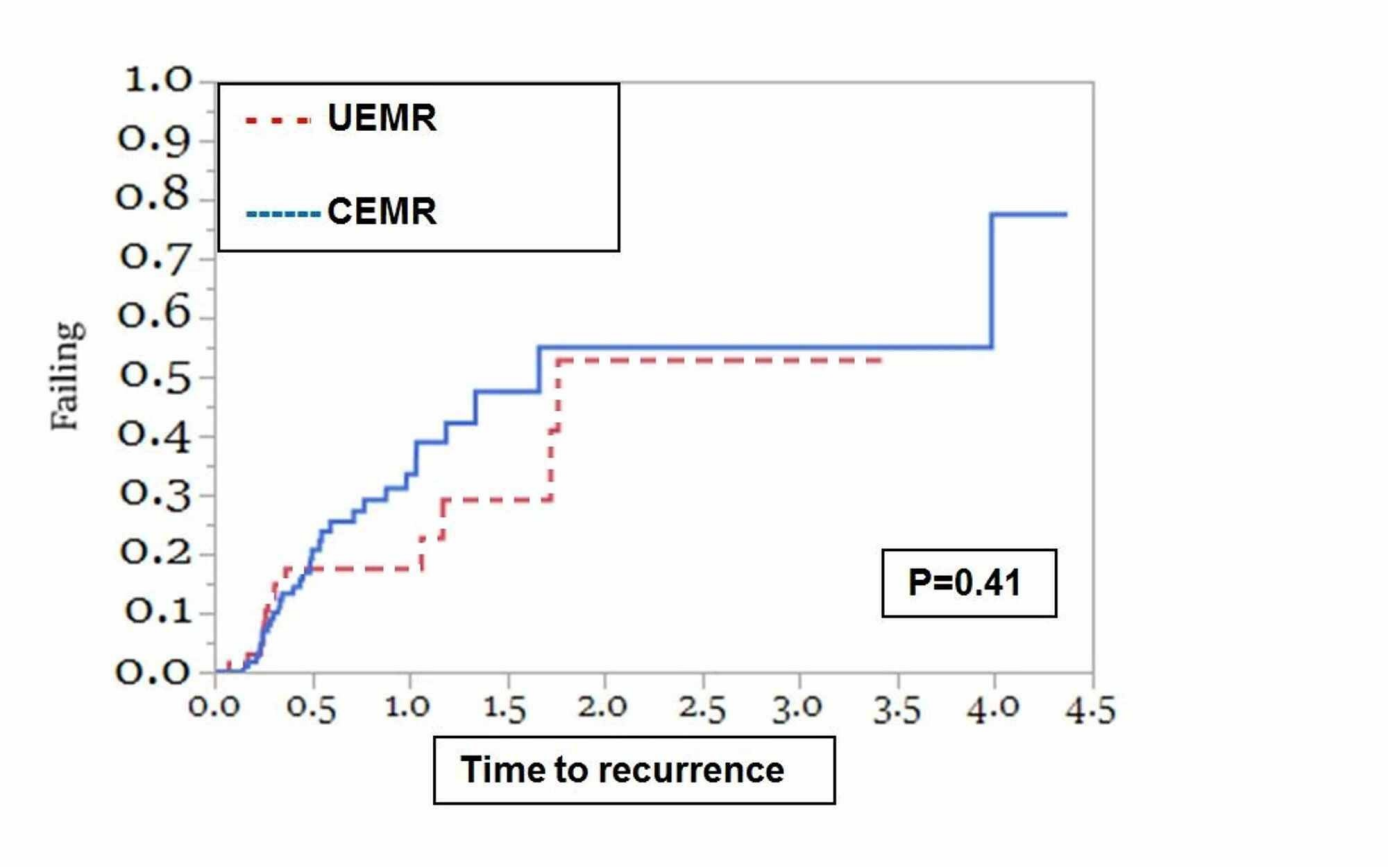
Polyp recurrence at the polypectomy site Kaplan-Meier curves for polyp recurrence among all patients who underwent UEMR and CEMR indicated there was no significant difference between the two groups. CEMR: conventional endoscopic mucosal resection; UEMR: underwater endoscopic mucosal resection

**Figure 3:**
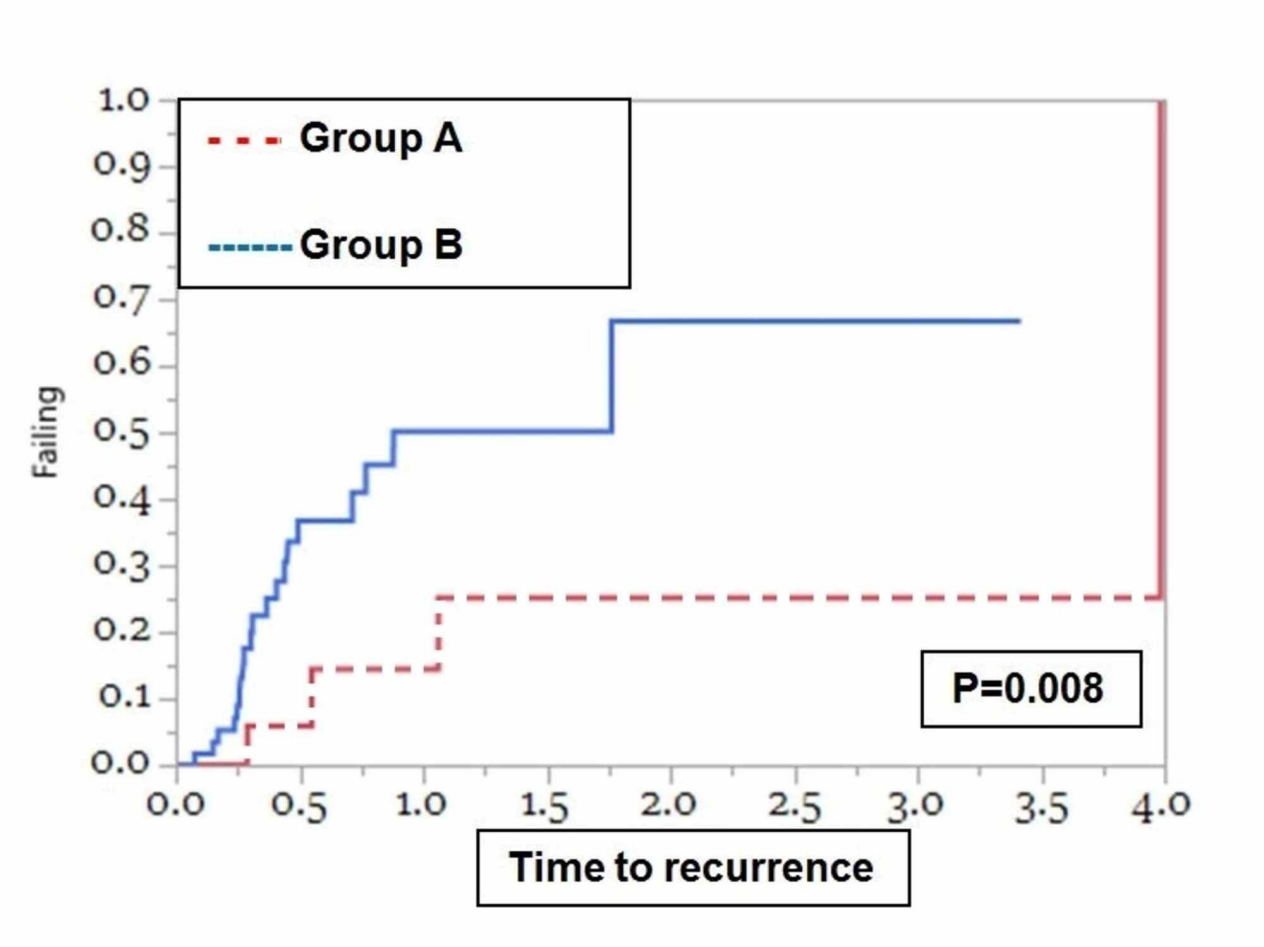
Polyp recurrence at the polypectomy site in patients undergoing piecemeal polypectomy Kaplan-Meier curves for polyp recurrence among patients who underwent piecemeal polypectomy (UEMR and CEMR groups) in two to three pieces (Group A) and piecemeal polypectomy (UEMR and CEMR) in more than three pieces (Group B) indicate that the median recurrence after polypectomy was shorter for patients in Group B as compared to those in Group A. CEMR: conventional endoscopic mucosal resection; UEMR: underwater endoscopic mucosal resection

Forty-six polyps recurred over a median follow-up of 0.48 years (IQR: 0.26 - 0.97). The histologies of the recurred polyps were tubular adenomas (23/46), villous adenomas (8/46), and sessile serrated adenomas (8/46). The rest (7/46) were traditional serrated adenomas and hyperplastic polyps. The cumulative incidence for polyp recurrence at three, six, and 12 months in the UEMR group was 6.5%, 17.5%, and 17.5%, respectively, and 7.0%, 19.3%, and 33.5% in the CEMR group, respectively. The most common site for recurrence was the ascending colon (39.0%), followed by the cecum (36.6%). Polyp recurrence occurred in 28/46 (60.9%) of the procedures done by faculty who did not complete advanced training. The mean recurrent polyp size was 2.17 ± 1.0 mm. About half of the polyps (24/46) that recurred were originally removed in a piecemeal fashion. The majority of polyps that recurred after en bloc resection (18/22) were removed using CEMR. The mean time for recurrence for polyps removed using the UEMR technique was not significantly different from polyps removed using the CEMR technique (1.42 ± 0.1 years vs. 2.25 ± 0.3 years; p = 0.41) (Figure 2). Since the majority of polyps resected were in the right colon, we compared the rates of recurrence in this region and found that the mean time for recurrence for right colon polyps removed using the UEMR technique by advanced endoscopists was significantly different from right colon polyps removed using CEMR by non-advanced endoscopists (1.42  ± 0.1 years vs. 0.84 ± 0.09 years; p = 0.01) (Figure 3).

Risk factors associated with recurrent polyps

By univariate analysis, polyp size (hazard ratio (HR) 1.22; 95% CI: 1.04 - 1.40, p = .02), degree of dysplasia (high/adenocarcinoma vs low/no dysplasia) (HR 2.88; 95% CI: 1.36 - 6.09, p = .02), completion of advanced training (yes vs no) (HR 0.38; 95% CI: 0.21 - 0.69, p < 0.01), and submucosal injection (HR 1.95; 95% CI: 1.04 - 3.66, p = .03) were all significant factors associated with polyp recurrence. In a multivariate model that included polyp size, degree of dysplasia, completion of advanced training, and submucosal injection, polyp size (HR = 4.64; 95% CI: 1.07 - 16.92, p = .04) and degree of dysplasia (high/adenocarcinoma vs low/no dysplasia) (HR = 2.40; 95% CI: 1.05 - 5.48, p = .04) were associated with increased risk for recurrence. However, completing advanced endoscopy training (HR = 0.41; 95% CI: 0.18 - 0.95, p = 0.04) was associated with decreased risk for recurrence (Table 2).

**Table 2:** Risk Factors for Polyp Recurrence at the Polypectomy Site CI: confidence interval

Risk Factors	Univariate Analysis Hazard Ratio (95% CI)	P	Multivariate Analysis Hazard Ratio (95% CI)	P
Polyp size (per mm)	1.22 (1.04 - 1.40)	0.015	4.64 (1.07 - 16.92)	0.041
Degree of dysplasia (high/cancer: no/low)	2.88 (1.36 - 6.09)	0.012	2.40 (1.05 - 5.48)	0.037
Polyp location (gastric/duodenum: colon)	0.51 (0.20 - 1.30)	0.124	-	-
Polyp location (right colon: left colon)	0.88 (0.58 - 1.34)	0.552		
Polyp shape (flat/sessile: pedunculated)	0.62 (0.24 - 1.57)	0.342	-	-
Number of polyps resected at polypectomy (per polyp)	0.62 (0.05 - 4.42)	0.664	-	-
Polypectomy device used (hot snare: cold snare)	2.03 (0.45 - 9.12)	0.358	-	-
Piecemeal removal (yes: no)	1.23 (0.69 - 2.20)	0.489	-	-
Submucosal lift (yes: no)	1.95 (1.04 - 3.66)	0.033	0.90 (0.42 - 1.92)	0.790
Completed advanced endoscopy training (yes: no)	0.38 (0.21 - 0.69)	0.002	0.41 (0.18 - 0.95)	0.037

Polyp recurrence after piecemeal resection for all polyps

**Figure 4:**
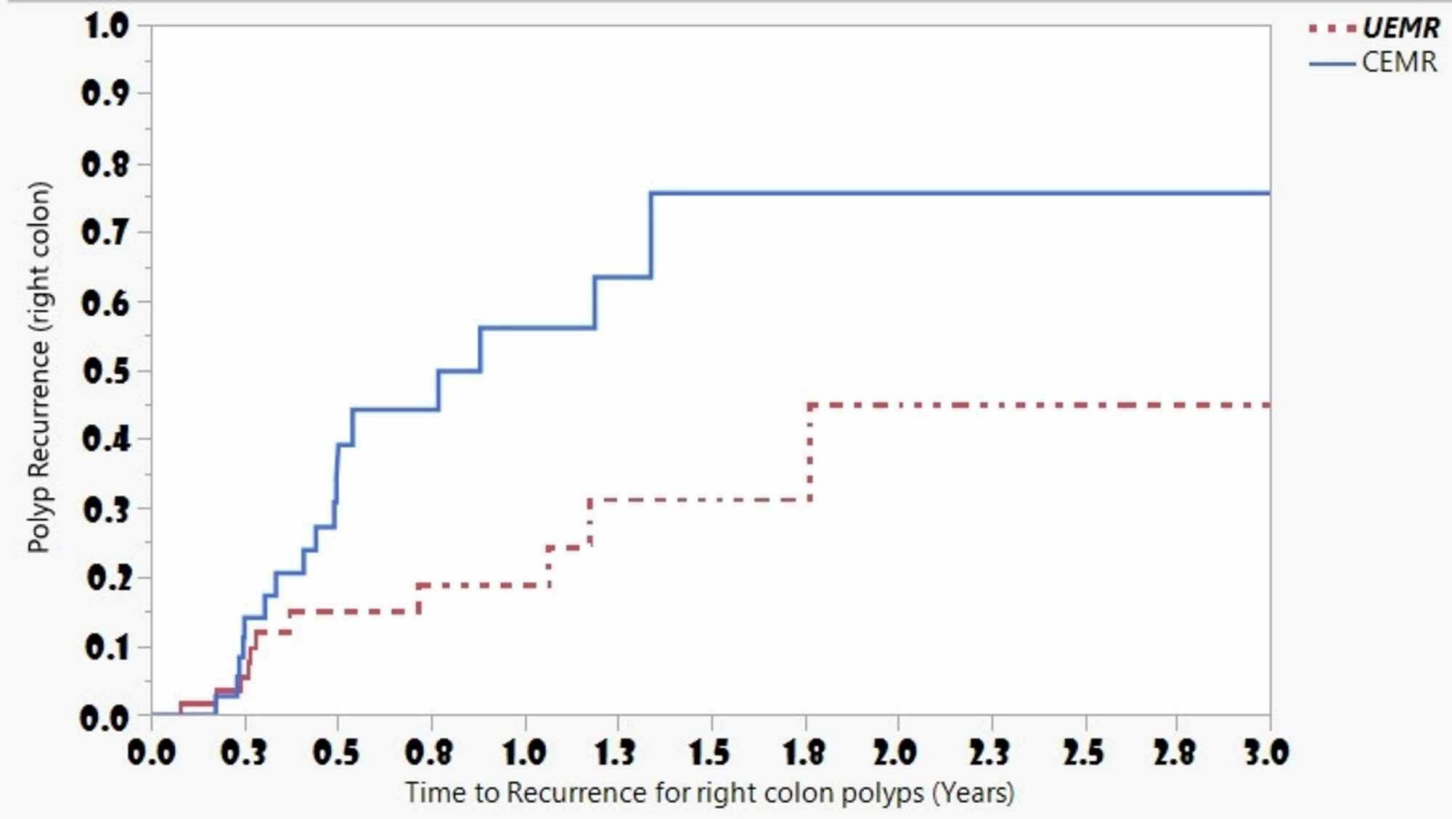
Polyp recurrence at the polypectomy site for right colon polyps Kaplan-Meier Curves for polyp recurrence among patients with right colon polyps who underwent UEMR (mainly done by advanced endoscopists) and CEMR (mainly done by non-advanced endoscopists) indicate that the median recurrence after polypectomy was shorter for polyps removed using CEMR. CEMR: conventional endoscopic mucosal resection; UEMR: underwater endoscopic mucosal resection

Eighty-nine patients (mean age: 63.7 ± 11.3 years; 48.3% males) with polyps removed using piecemeal EMR (31 (34.8%) removed in two or three pieces (Group A) and 58 (65.2%) removed in more than three pieces (Group B) were identified. Most of the polyps resected by piecemeal EMR were located in the right colon 61/89 (68.5%) and 43/89 (48.3%) were treated with a submucosal injection. At follow-up endoscopy, four of the 31 (12.9%) patients in Group A and 20 of the 58 (34.5%) patients in Group B developed polyp recurrence at the polypectomy site. The median recurrence after polypectomy was longer for patients in Group A as compared to those in Group B (3.98 years vs. 0.88 years, p = 0.008) (Figure 4). A subgroup analysis comparing polyp resection using en bloc or piecemeal fashion between CEMR and UEMR did not show any statistically significant difference between the two groups (figures were not included). 

## Discussion

This study showed that UEMR is a safe and effective technique to remove large gastrointestinal polyps. Polyps recurred in 19.1% of the resected polyps, which is higher than what has been previously reported [10].

Tubular adenomas were the most commonly observed histology among the polyps that recurred, followed by villous and sessile serrated adenomas. About one-half of patients who underwent en bloc resection had a recurrent polyp; most of them were removed using the CEMR technique. This could be secondary to lesion displacement during CEMR [14]. Polyp recurrence occurred earlier in patients undergoing piecemeal resection of more than three pieces. We found that increasing polyp size, degree of training, and degree of dysplasia were associated with increased polyp recurrence.

In our study, we confirmed the finding by Schenck et al. that the recurrence rate after polypectomy at the first follow-up colonoscopy was greater for CEMR compared to UEMR [10]. The rate of recurrence after CEMR in our study is comparable to the published rate of recurrence, which ranges from 15% to 50% [10, 15-22], but was higher in the UEMR group than what was reported in the literature. This is likely because we included patients who had undergone prior attempted resection which is reported to have higher recurrence rates, and we also included patients with difficult locations, such as ileocecal polyps, that previous studies excluded [9, 23]. Although it is known from previous studies that piecemeal EMR is associated with more recurrent adenomatous tissues as compared to en bloc EMR, we did not find a difference in the recurrence rate between patients undergoing piecemeal resection vs. en bloc resection [16-17]. En bloc resection with CEMR accounted for most of the recurrences compared to UEMR. This suggests that UEMR provided better visualization of the resection bed and less lesion displacement in comparison to CEMR. Our data also showed that the majority of the polyps that recurred were tubular and villous adenomas and located in the right colon, both of which were found to be risk factors for recurrence in previous studies [24].

In our study, we also confirmed the finding by Sakamoto et al. that patients undergoing piecemeal EMR which involved five or more specimens were associated with a greater rate of local recurrence [20]. We used a cut-off of three pieces instead of five pieces and found that the likelihood of local recurrence was about three times greater in the group with more resected pieces. Our piecemeal resection rate was lower than what was published in previous studies [10, 19]. However, this could be because our advanced endoscopists received dedicated EMR training and we also included polyps smaller than 2 cm which have a lower rate of piecemeal resection [25].

Our study highlights the risk of polyp recurrence at a surveillance endoscopy for the follow-up of an index polyp removed using CEMR and UEMR. Many studies have evaluated clinical and endoscopic predictors for recurrence [24, 26-27]. Our study showed a clear association between polyp size and degree of dysplasia and polyp recurrence, supporting what previous studies have consistently shown [19]. We also evaluated the degree of the performing provider’s training as a risk factor for recurrence after EMR and found that providers who completed advanced endoscopy training had lower recurrence rates compared to ones who did not. This factor was not studied in the past as a risk factor for recurrence. Some factors, such as the endoscopist’s experience and adenoma detection rate (ADR), were reported to predict the recurrence rate after advanced polyps at surveillance [28]. Our findings of a low recurrence rate could be explained by the fact that some advanced endoscopists in our practice have very high ADR (about 50%). Although one study with a small sample size suggested that an ADR of 37% is adequate for high-quality surveillance examinations [29], further studies are warranted to evaluate the role of ADR and the endoscopist’s degree of training in polyp recurrence after polypectomy.

Although the UEMR technique does not require submucosal injection, it was required about 15% of the time. This could be because the performing provider performed hybrid resection using UEMR after submucosal injection. Although we did not encounter any perforation in our studied population, one should be careful since colonic wall stretching by the submucosal injection was blamed to be the cause of perforation in one reported case [12].

In our series, delayed bleeding was not significantly different between the two techniques. A delayed bleeding rate of 4.4% is similar to what was reported by Binmoeller et al. [9]. Hot snare use in the majority of EMR cases could be to blame since delayed bleeding was reported to be significantly higher after hot polypectomy [30].

Our study has some limitations. In addition to the retrospective nature of the study, we were not able to obtain data on all patients who had surveillance endoscopy done somewhere else. Another limitation was the relatively low number of upper gastrointestinal polyps, pedunculated polyps, and sessile serrated adenomas since the sample size was small. This is due to the fact the UEMR is a new technique and many of the cases with en bloc resection and no dysplasia have not had a repeat examination yet. We could not report if the edges were cauterized or not which is another weak point. We could not use ADR in our risk analyses since many advanced endoscopists in our practice do not do screening colonoscopy very often and it is difficult to estimate their ADR.

## Conclusions

In conclusion, UEMR is less technically difficult than CEMR. It is associated with better identification of polyp margins and a lower recurrence rate than CEMR in right colon polyps. Randomized controlled trials are recommended to compare both procedures to better stratify a polyp’s rate and risk for recurrence.

## References

[1] Haggar FA, Boushey RP (2009). Colorectal cancer epidemiology: incidence, mortality, survival, and risk factors. Clin Colon Rectal Surg.

[2] Vogelstein B, Fearon ER, Hamilton SR (1988). Genetic alterations during colorectal-tumor development. N Engl J Med.

[3] Zauber AG, Winawer SJ, O'Brien MJ (2012). Colonoscopic polypectomy and long-term prevention of colorectal-cancer deaths. N Engl J Med.

[4] Nishihara R, Wu K, Lochhead P (2013). Long-term colorectal-cancer incidence and mortality after lower endoscopy. N Engl J Med.

[5] Yokota T, Sugihara K, Yoshida S (1994). Endoscopic mucosal resection for colorectal neoplastic lesions. Dis Colon Rectum.

[6] Mouchli MA, Ouk L, Scheitel MR (2018). Colonoscopy surveillance for high risk polyps does not always prevent colorectal cancer. World J Gastroenterol.

[7] Conio M (2011). Endoscopic mucosal resection. Gastroenterol Hepatol (N Y).

[8] Martínez ME, Sampliner R, Marshall JR, Bhattacharyya AK, Reid ME, Alberts DS (2001). Adenoma characteristics as risk factors for recurrence of advanced adenomas. Gastroenterology.

[9] Binmoeller KF, Weilert F, Shah J, Bhat Y, Kane S (2012). Underwater EMR without submucosal injection for large sessile colorectal polyps (with video). Gastrointest Endosc.

[10] Schenck RJ, Jahann DA, Patrie JT (2017). Underwater endoscopic mucosal resection is associated with fewer recurrences and earlier curative resections compared to conventional endoscopic mucosal resection for large colorectal polyps. Surg Endosc.

[11] Sandhu DS, Lee YJ, Gerke H (2018). Underwater endoscopic mucosal resection: an alternative treatment for large colorectal polyp removal. Minerva Gastroenterol Dietol.

[12] Kawamura T, Sakai H, Ogawa T (2018). Feasibility of underwater endoscopic mucosal resection for colorectal lesions: a single center study in Japan. Gastroenterology Res.

[13] Ponugoti PL, Rex DK (2016). Perforation during underwater EMR. Gastrointest Endosc.

[14] Siau K, Ishaq S, Cadoni S, Kuwai T, Yusuf A, Suzuki N (2018). Feasibility and outcomes of underwater endoscopic mucosal resection for ≥ 10 mm colorectal polyps. Surg Endosc.

[15] Tajika M, Niwa Y, Bhatia V (2011). Comparison of endoscopic submucosal dissection and endoscopic mucosal resection for large colorectal tumors. Eur J Gastroenterol Hepatol.

[16] Moss A, Williams SJ, Hourigan LF (2015). Long-term adenoma recurrence following wide-field endoscopic mucosal resection (WF-EMR) for advanced colonic mucosal neoplasia is infrequent: results and risk factors in 1000 cases from the Australian Colonic EMR (ACE) study. Gut.

[17] Belderbos TD, Leenders M, Moons LM, Siersema PD (2014). Local recurrence after endoscopic mucosal resection of nonpedunculated colorectal lesions: systematic review and meta-analysis. Endoscopy.

[18] Khashab M, Eid E, Rusche M, Rex DK (2009). Incidence and predictors of "late" recurrences after endoscopic piecemeal resection of large sessile adenomas. Gastrointest Endosc.

[19] Buchner AM, Guarner-Argente C, Ginsberg GG (2012). Outcomes of EMR of defiant colorectal lesions directed to an endoscopy referral center. Gastrointest Endosc.

[20] Sakamoto T, Matsuda T, Otake Y (2012). Predictive factors of local recurrence after endoscopic piecemeal mucosal resection. J Gastroenterol.

[21] Friedland S, Banerjee S, Kochar R, Chen A, Shelton A (2014). Outcomes of repeat colonoscopy in patients with polyps referred for surgery without biopsy-proven cancer. Gastrointest Endosc.

[22] Knabe M, Pohl J, Gerges C, Ell C, Neuhaus H, Schumacher B (2014). Standardized long-term follow-up after endoscopic resection of large, nonpedunculated colorectal lesions: a prospective two-center study. Am J Gastroenterol.

[23] Kim HG, Thosani N, Banerjee S, Chen A, Friedland S (2014). Underwater endoscopic mucosal resection for recurrences after previous piecemeal resection of colorectal polyps (with video). Gastrointest Endosc.

[24] Briedigkeit A, Sultanie O, Sido B, Dumoulin FL (2016). Endoscopic mucosal resection of colorectal adenomas > 20 mm: risk factors for recurrence. World J Gastrointest Endosc.

[25] Raju GS, Lum PJ, Ross WA (2016). Outcome of EMR as an alternative to surgery in patients with complex colon polyps. Gastrointest Endosc.

[26] Vinsard DG, Kandel P, Mejia Perez LK (2018). Adenoma recurrence after endoscopic mucosal resection: propensity score analysis of old and new colonoscopes and Sydney recurrence tool implementation. Endosc Int Open.

[27] Jang ES, Kim JW, Jung YJ (2013). Clinical and endoscopic predictors of colorectal adenoma recurrence after colon polypectomy. Turk J Gastroenterol.

[28] Hassan C, Repici A, Rex DK (2018). Post-polypectomy recurrence: low detector or high-risk polyp?. United European Gastroenterol J.

[29] Anderson JC, Butterly LF, Goodrich M, Robinson CM, Weiss JE (2013). Differences in detection rates of adenomas and serrated polyps in screening versus surveillance colonoscopies, based on the new hampshire colonoscopy registry. Clin Gastroenterol Hepatol.

[30] Yamashina T, Fukuhara M, Maruo T (2017). Cold snare polypectomy reduced delayed postpolypectomy bleeding compared with conventional hot polypectomy: a propensity score-matching analysis. Endosc Int Open.

